# Cost-effectiveness of providing patients with information on managing mild low-back symptoms in an occupational health setting

**DOI:** 10.1186/s12889-016-2974-4

**Published:** 2016-04-12

**Authors:** J. Rantonen, J. Karppinen, A. Vehtari, S. Luoto, E. Viikari-Juntura, M. Hupli, A. Malmivaara, S. Taimela

**Affiliations:** Finnish Institute of Occupational Health (FIOH), P.B. 40, 00251 Helsinki, Finland; Helsinki Institute for Information Technology HIIT, Department of Computer Science, Aalto University, Espoo, Finland; Department of Physical Medicine and Rehabilitation, South Karelia Social and Health Care District, Lappeenranta, Finland; Medical Research Center Oulu, University of Oulu and Oulu University Hospital, Oulu, Finland; National Institute for Health and Welfare, Centre for Health and Social Economics, Helsinki, Finland; Evalua International, Espoo, Finland; Hjelt Institute, Department of Public Health, University of Helsinki, Helsinki, Finland; South Karelian Institute, Lappeenranta University of Technology, Lappeenranta, Finland; University of Helsinki, Helsinki, Finland

**Keywords:** Low back pain, The Back Book, Prevention, Quasi-experimental study, Sickness absence, Intervention, Disability, Cohort study, RCT, Health economy

## Abstract

**Background:**

Evidence shows that low back specific patient information is effective in sub-acute low back pain (LBP), but effectiveness and cost-effectiveness (CE) of information in early phase symptoms is not clear. We assessed effectiveness and CE of patient information in mild LBP in the occupational health (OH) setting in a quasi-experimental study.

**Methods:**

A cohort of employees (*N* = 312, aged <57) with non-specific, mild LBP (Visual Analogue Scale between 10–34 mm) was selected from the respondents of an employee survey (*N* = 2480; response rate 71 %). A random sample, representing the natural course of LBP (NC, *N* = 83; no intervention), was extracted as a control group. Remaining employees were invited (181 included, 47 declined, one excluded) into a randomised controlled study with two 1:1 allocated parallel intervention arms (“Booklet”, *N* = 92; “Combined”, *N* = 89). All participants received the “Back Book” patient information booklet and the Combined also an individual verbal review of the booklet. Physical impairment (PHI), LBP, health care (HC) utilisation, and all-cause sickness absence (SA) were assessed at two years. CE of the interventions on SA days was analysed by using direct HC costs in one year, two years from baseline. Multiple imputation was used for missing values.

**Results:**

Compared to NC, the Booklet reduced HC costs by 196€ and SA by 3.5 days per year. In 81 % of the bootstrapped cases the Booklet was both cost saving and effective on SA. Compared to NC, in the Combined arm, the figures were 107€, 0.4 days, and 54 %, respectively. PHI decreased in both interventions.

**Conclusions:**

Booklet information alone was cost-effective in comparison to natural course of mild LBP. Combined information reduced HC costs. Both interventions reduced physical impairment. Mere booklet information is beneficial for employees who report mild LBP in the OH setting, and is also cost saving for the health care system.

**Trial registration:**

ClinicalTrials.gov NCT00908102

**Electronic supplementary material:**

The online version of this article (doi:10.1186/s12889-016-2974-4) contains supplementary material, which is available to authorized users.

## Background

Low back pain (LBP) is a common health problem that often leads to primary health care attention [1]. In the global scale, LBP is still the leading cause of disability and sickness absence among the workforce, yet inducing a huge impact on the economy [1–3]. Obviously, there is a need for innovative, cost-effective methods for managing LBP and for preventing the associated disability [4, 5].

Patient information can help patients understand and cope with their medical conditions and it may provide reassurance as regards their prognosis [6–10]. In order to promote efficient self-care, the content should be evidence-based or at least concurrent with existing guidelines [6, 11–13]. The optimal patient group and the type of information (personal or group, oral or written etc.) should also be determined. Moreover, it should be decided who is the main responsible person for the delivery of the information [14]. Educational booklets have been used in mediating general patient information, either alone or combined with, for example, personal verbal advice or educational group sessions [14]. The Back Book is probably the most widely used guideline-based patient information booklet for LBP [8, 13, 15–18].

In 2012, approximately 1.85 million Finnish employees (86 % of the total workforce) were covered by occupational health (OH) service. Besides 1.1 million health examinations, OH also performed 5.2 million illness-related visits [19]. Because most employees use OH service for all their primary health care (HC) needs, Finnish OH professionals are continually facing the challenge of how to manage employees’ LBP-related disability [20–22].

Recently, we adopted the Back Book (booklet) based patient information for the self-management of mild (low-risk) LBP patients. The booklet alone was as effective in LBP-specific outcomes and sickness absence (SA) as the combination of the booklet and face-to-face verbal information in a randomized trial [15]. However, the feasibility of patient information was not fully assured since there was no comparison to the natural course of LBP in the previous trial.

In the present study we have assessed the clinical effectiveness and cost-effectiveness (CE) of providing booklet based patient information in comparison to the natural course of LBP (no intervention) on mild LBP in a longitudinal, quasi-experimental intervention trial in the OH setting. Direct health care (HC) costs were compared to all-cause SA in the CE analysis.

## Methods

### Study design and ethics

The original study design was a longitudinal cohort study with two embedded RCTs and a control group that underwent no intervention (natural course of LBP). The detailed study design and results of both RCTs have been published elsewhere [15, 23].

All employees (*N* = 2480) in a forestry company were invited to respond to a postal survey on LBP and related PHI during September 2001. On the basis of their responses (*N* = 1754, response rate 71 %), employees were allocated into three main categories: “no” low back (LB) symptoms, “some” LB symptoms i.e. mild LBP (RCT1), and LB symptoms that “potentially hamper work” i.e. moderate LBP (RCT2). The main results of both RCTs have already been published. RCT2 showed that two multidisciplinary and active interventions reduced LBP, sickness absence and physical impairment among employees who were fit to work but reported moderate level LBP [23]. RCT1 showed that among employees who reported mild LBP (but were still able to work), booklet based patient information was as effective (in terms of clinical effectiveness) as additional face-to-face verbal advice with the booklet [15].

**Fig. 1:**
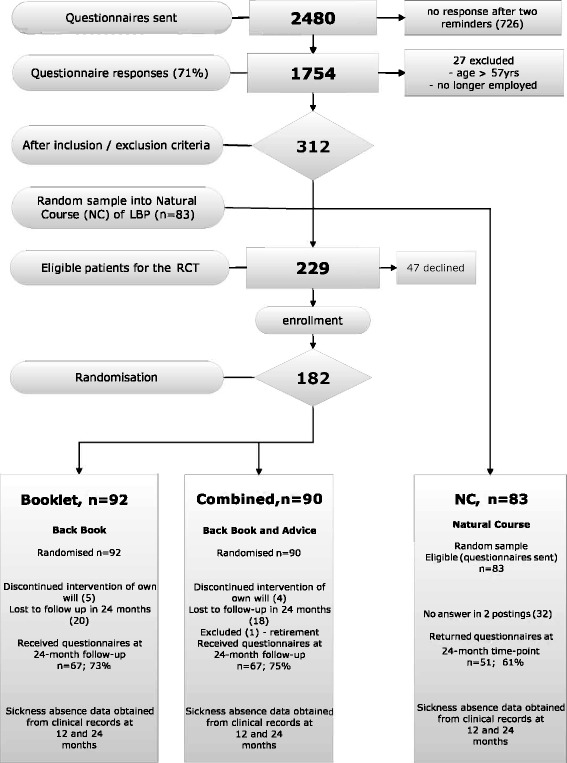
Flow diagram of the course of the study, showing the number of participants at different phases of the trial [RCT, randomized controlled trial]

In this paper, we will focus on employees that reported some LBP symptoms, defined as mild LBP [24]. Alongside our previous RCT1 [15], we analysed the clinical effectiveness and cost-effectiveness of two patient information strategies in comparison with the natural course of LBP (no intervention), two years after the employee survey. Figure 1 shows the flow chart of the study participants at different stages of the intervention trial.

Throughout the study, information about the study procedures and the management of LBP were shared in collaboration with all stakeholders of the company (e.g. employer, employees, study personnel, trade union representatives and workers’ compensation board).

The South Karelian Central Hospital Research Ethics Board approved the study, which was performed according to the Declaration of Helsinki.

### Study population

Employees were eligible for the study, if they reported (in their responses to the postal questionnaire) LBP intensity between 10 and 34 mm on a 100-mm Visual Analogue Scale (VAS) during the past week, were aged under 57, and fulfilled at least one of the following criteria:

1. LBP lasting two weeks or more in the previous 12 months
2. LBP radiating below knee level at the time of responding to the questionnaire
3. Recurrent LBP (two or more episodes during the previous 12 months, irrespective of their duration)
4. Self-reported work absence due to LBP during the previous 12 months

Exclusion criteria were: retirement within the time span of the follow-up, pregnancy, presence of acute nerve root compression symptoms, suspicion of malignant tumours, recent spine fracture, severe osteoporosis or any other disease that prevented participation in the follow-up.

Sickness absence at the inclusion phase of the study was neither an inclusion nor an exclusion criterion for the study. Initially, employees were able to work regardless of their previous or present symptoms (whether LB related or other).

Eligible employees (*n* = 312) were defined as a mild LBP cohort. Since there is no gold standard for distinguishing mild LBP from moderate symptoms, the pain limits were chosen arbitrarily. Pain categorisation follows, for example, the recommendations of Boonstra et al. [24], who has categorised mild pain as VAS ≤34 mm, moderate pain as between 35 and 74 mm, and severe pain as ≥ 75 mm.

Prior to the invitation to participate in the intervention, a random sample of employees (83) was extracted from the cohort in order to form a natural course (NC) control group. The remaining employees (*n* = 229) were invited to visit an OH nurse, who was specially trained in LBP problems and the main study procedures. A total of 182 employees were randomised (47 declined to participate) into two intervention arms, Booklet (Back Book) and Combined (Back Book and Advice), but soon after randomisation, one person was excluded from the Combined group due to retirement. Therefore, finally 181 participants (Booklet, *n* = 92 and Combined, *n* = 89) were included in the intervention groups. Of the 83 employees that were eligible for NC, 51 responded to the postal questionnaire, resulting in missing data for 32 employees.

Table 1 shows the distribution of some basic characteristics (employee survey data) in the study groups. Throughout the study, all participants were able to use HC services as usual, either at OH services or other available HC units.

**Table 1:** Basic characteristics of the study participants

Characteristics	Combined (*n* = 89)			Booklet (*n* = 92)			NC (*n* = 83)			*p*
	%	mean	SD	%	mean	SD	%	mean	SD	
Demographics										
Age (years)	.	44	8	.	43	7	.	45	8	0.52
Male	79	.	.	66	.	.	76	.	.	0.55
Smoking	30	.	.	28	.	.	31	.	.	0.35
High school diploma/vocational degree	79	.	.	75	.	.	76	.	.	0.87
General health										
Duration of LBP, years	.	12	9	.	11	7	.	14	9	0.09
SA days before baseline^a^					9	12		14	19	0.10
Work-related features										
Blue collar worker	69	.	.	64	.	.	78	.	.	0.05
Shift worker (two or three shift work)	41	.	.	37	.	.	40	.	.	0.73
Physical workload (1–5)^b^	.	3	1	.	3	1	.	3	1	0.13
Mental workload (1–5)^b^	.	3	1	.	3	1	.	3	1	0.51
Work ability (0–10)^c^	.	8.1	1.5	.	8.3	1.5	.	7.8	1.6	0.07
Outcome variables at baseline										
Physical impairment (PHI); RM-18 (0–18)^d,e^	.	4.2	4.6	.	2.5	3.2	.	3.9	3.6	0.34
Low back pain (LBP); VAS (0–100)^d^, mm	.	20	7	.	20	7	.	19	7	0.52
Other LB specific variables										
Fear Avoidance Beliefs Questionnaire (13–78)^d^	.	29	10	.	29	11	.	31	11	0.15

^a^all cause sickness absence days during 12 months prior to baseline (register data)

^b^1–5 indicates self-rated load: 1 = very heavy, 2 = moderate, 3 = intermediate, 4 = rather light, 5 = very light

^c^range 0–10, when 0 is lowest possible work ability and 10 is best possible work ability

^d^Higher value indicates higher impairment, pain or fear of pain, respectively

^e^for the comparison between Booklet and NC, mean difference of PHI is significant (*p* = 0.01)

Means (SD = standard deviation) or percentages when applicable. Intervention groups were pooled for the comparison between the intervention and NC. [Combined = Back Book and Advice intervention group; Booklet = Back Book intervention group; *NC* Natural Course control group; *BMI* Body mass Index; *SA* sickness absence; *LBP* low back pain; *VAS* Visual Analogue Scale; *RM-18* Roland-Morris 18-item Disability Questionnaire; *PHI* Physical impairment;; *p* = *P*-value]. Missing values (concerning smoking, duration of LBP and shift work) were imputed with the multiple imputation procedure

### Randomisation and blinding

The NC group was extracted from the mild LBP cohort as a random sample by means of a computer program. Remaining employees were invited to the first study visit.

The randomisation scheme for the trial was prepared by an independent biostatistician before the study was started by using a computer-generated randomisation table with a block size of eight. A research assistant prepared the sealed envelopes that included the group assignments, referral either to the Booklet or the Combined group.

During the first study visit, OH nurse informed the employee about the study, collected the informed consent, performed a clinical examination and explained the findings to the employee. After that, she opened the sealed envelope that included the assignment for either of the two interventions, according to the randomisation scheme. More details of the original RCT are explained elsewhere [15]. In order to ensure blinded analysis, all study data were entered into the data file by persons who were not related to the study.

### Interventions

The company OH services unit operated as usual during the study period, and information about the study was provided regularly in the personnel magazine of the company and intranet.

At the end of the first study visit, the OH nurse gave the Back Book (booklet) to all participants and presented additional face-to-face oral review of the booklet into those participants who were randomised into the Combined arm.

The randomisation visit lasted about 60 min in the Booklet group, whereas in the Combined group, the additional review of the booklet, face-to-face with the employee required 20 min more.

Follow-up visit intervals were similar in both intervention groups, scheduled at 3, 6, 12, and 24 months from the first visit. During scheduled visits, OH nurse checked the returned questionnaire responses, performed a simple clinical balance test, and answered questions that may have arisen among the participants. Generally, the follow-up questionnaires had been filled in by the employees during the week prior to the visit date. Both intervention groups were comparable as regards the follow-up intervals, visit activity and the time spent (30 min) at the follow-up visits.

NC group received a postal questionnaire, similar to those addressed to the intervention groups and scheduled at 24 months from the start of the whole study. For this paper, we have analysed the 24-month questionnaire data, because it is comparable between the intervention groups and NC.

#### Booklet (*n* = 92)

Participants received a personal copy of the booklet. They underwent no other intervention.

The information in the booklet is based on the bio-psychosocial model and focuses on attitudes and inappropriate behaviour concerning LBP. The booklet has been designed to complement and support evidence-based LBP management strategies, and its key messages follow the current national guidelines. It also includes information on how to cope with LBP, avoid re-exacerbation of LBP, and emphasises that one should reassume normal activities and return to work as soon as possible. The book has been translated into Finnish from the original English version [8].

#### Combined (*n* = 89)

Participants in the Combined group received the booklet during their first visit, but the contents of the booklet were also reviewed to them face-to-face by the OH nurse.

#### NC (*n* = 51)

Participants in the NC group were not invited to study visits, but the study questionnaire was sent to them 24 months after the employee survey. Non-respondents of the first postal questionnaire were sent one reminder soon after the first one. Of the 83 eligible employees, 51 (61 %) responded to the questionnaire and were included in the complete case analysis.

### Use of health care (HC) resources

About 24 months after the employee survey (i.e. baseline), participants in all study groups were asked to report their HC utilisation during the previous 12 months, i.e. 13–24 months after the baseline. The questionnaire included the following items: the number of visits to a physician, nurse, physiotherapist or other HC professional; each representing a unit of HC utilisation. These items were included in the following HC categories: OH, public (primary) HC, private HC, hospital outpatient clinics, and hospital inpatient care and rehabilitation clinics. The number of radiological procedures during the previous 12 months and the visits that relate to alternative or complimentary HC (acupuncture, massage, chiropractor etc.) were also included. Our HC usage data shows separately the visits in the OH, public HC and private sector, but they all may be considered as primary care visits. Service-wise the contents of care are comparable. Because their unit costs vary, we have presented them separately. The unit costs were obtained from the national working paper of the Finnish Ministry of Social Affairs and Health [25], expressed in euros, and converted to the 2004 level (the final follow-up visit was in 2004). Additional table shows the use of HC resources over 12 months, scheduled at 13–24 months from the baseline (see Additional file 1).

We have focused on the direct HC costs and considered SA as the primary outcome of the CE analysis. Travelling costs and productivity losses (i.e. employee not working in his/her working time) were not included in the costs. All study participants were working at the same industrial complex area. Intervention cost was evaluated with the extra time consumed for the verbal patient information in the OH nurse visit (20€/person). In proportion to the 12-month HC costs, we also collected all cause SA days from 12 months into our cost-effectiveness analysis (CEA). Cost-effectiveness (CE) was evaluated from the health care perspective and the HC usage as well as the SA data in CEA covered the same time period (12 months, timeline of 13–24 months from baseline).

### Health outcomes

Baseline measurements originate from the employee survey. The follow-up measurements were performed 24 months after the baseline. Time interval is the same in all study groups. Clinical effectiveness was evaluated with the following variables: physical impairment (PHI), intensity of LBP and accumulated, all cause sickness absence (SA). PHI was measured with the Roland-Morris Disability Questionnaire 18-item scale [26, 27], and LBP by the Visual Analogue Scale (VAS; 0–100 mm) [28]. SA data were gathered at one-year intervals up to two years after the individual randomisation date of the participants in the intervention groups. As regards the NC group, SA count started from the postal date of the employee survey. SA data is comprehensive and highly reliable because it is based on the administrative payroll system of the employer. There were no missing values in the SA data, either. Since all SA episodes in the OH service’s administrative registration system included ICD-10 [29] classification numbers, we were able to distinguish LB specific SA from the other cause SA episodes. Although the total (=all cause) SA days were included in the final analyses, each SA episode that was not LB-specific and at the same time longer than 30 days was omitted from the analysis in order to prevent severe diseases (other than LB specific) and sequels of injuries (other than LB specific) from interfering with the analysis. Therefore, SA data includes all LB specific SA episodes, regardless of their length, and all other cause SA episodes that are less than 31 days. The cut-off limit of 30 days per SA period was chosen arbitrarily.

### Power calculations

In the two-arm randomised trial the power calculations were based on the following assumptions: the estimated standard deviation for the PHI score in our population was 4 units. A difference of 2 units between treatment arms is detectable with 85 % power in two-tailed tests with the alpha of 0.05 for a sample of 73 employees in each group, and the standardised effect size is 0.50.

### Statistical analyses

All statistical analyses were performed at employee level, according to the intention-to-treat principle. About 29 % of the data concerning HC costs and health outcomes were missing, mostly due to missed follow-up visits. However, all participants in this study had an equal opportunity to attend the follow-up visits or send their questionnaire information to the study personnel. Therefore, we have assumed that the missing data is missing at random. All missing data were imputed with the multiple imputation method [30] by using the SPSS Statistical Package version 22.0 for Windows ® (SPSS Inc., Chicago, IL, USA).

The following items were either used as determinants or were also imputed (if used with outcomes or characteristics) in the multiple imputation procedure: age, gender, marital status, education, smoking, lifetime duration of LBP, self-assessed health status, working status, shift work, physical workload, mental workload, self-assessed work ability, job satisfaction, physical impairment, LBP, pain related fear, all cause SA 12 months prior to employee survey, all cause SA in the first follow-up year.

We have presented our results from the basis of two databases, the complete case (original) data and the imputed data (main analysis). Basic characteristics were compared using descriptive statistics. Intervention groups were pooled for the comparison.

The effectiveness of an intervention was primarily estimated by the group difference (Combined vs. NC; Booklet vs. NC) of the outcome variable (PHI and LBP) after two years. For the clinical effectiveness analysis, SA days were accumulated in two years from the baseline. Statistical analyses of the clinical outcomes were performed with the SPSS 22.0 for Windows.

For the cost-effectiveness analyses, SA data were gathered in 13–24 months from the baseline in order to comply with the timeframe of HC costs in an equal way in all study groups. Typically, SA distributions were skewed.

Some of the total HC usage data required manual corrections before transmission into the computer database. For instance, the costs of some radiological tests were accounted manually because of variable unit costs of the different tests.

In order to assess the uncertainty in CEA, we performed a one-way sensitivity analysis and a probabilistic sensitivity analyses (Monte Carlo method and Bayesian, non-parametric bootstrapping with 10 000 replicates) of the comparisons between the intervention groups and NC.

Incremental cost-effectiveness ratio (ICER) is the cost-difference of two interventions divided with the difference of their effects. Hence, ICER summarizes the cost-effectiveness of an HC intervention by representing the average incremental cost (€) which associates in one additional unit of effect (SA day).

One way sensitivity analysis shows how the change in one unit cost influences in the ICER (€ per sickness absence day), when other values remain at their base level.

The results of the CEA main analysis are presented as CE-planes, mean incremental costs (IC) and effects (IE) with corresponding 95 % confidence intervals (95%CI) and the ICER. The uncertainty of CE planes was evaluated with cost-effectiveness acceptability curves (CEAC), which are presented in the additional files. One-way sensitivity analyses for the ICERs are presented in tornado diagrams. The results of the imputed main analysis have also been compared to the complete case analysis (original data).

### Lost to follow-up

Within the first three months, four participants from the Combined and five from the Booklet group left the study due to personal reasons, but granted permission to use their data. At the end of the two-year follow-up, 18 participants from the Combined, and 20 participants from the Booklet failed to return their questionnaires, resulting in missing data. The reasons for not continuing with the study remained mostly unknown to the researchers. In both intervention groups, 67 participants continued to the end of the two-year follow-up (activity rates: Combined 73 % and Booklet 75 %). In the NC group, 32 of the eligible 83 persons did not return the postal questionnaire, meaning that the complete data is available for 51 (61 %) participants.

Complete case analysis includes only those participants who returned their HC utilisation questionnaires in the 24-month visit. In the main analysis, however, SA data (as also the multiply imputed questionnaire data) were analysed among all study participants.

## Results and discussion

### Use of HC resources

According to the reported HC usage (see Additional file 1), the direct HC cost per person in the Combined (*n* = 67), Booklet (*n* = 67) and NC (*n* = 51) groups was 188€ (range: 0 – 6543€; quartiles: 20, 20, 77€), 73€ (range: 0–773€; quartiles: 0, 0, 65€) and 370€ (range: 0–3379€; quartiles: 0, 0, 387€), respectively. The corresponding sums for the three groups were 12 580€, 4880€ and 18 863€ per year.

### Cost effectiveness (main) analysis (CEA)

Using the imputed cost data (main analysis) of 264 participants, the Booklet intervention was less costly and more effective than NC in a timeframe of 13–24 months after baseline. Also the Combined intervention reduced HC costs, but the effectiveness was only modest. The ICER in Booklet vs. NC was 54€ and in Combined vs. NC 315€ meaning the amount of money required for each avoided SA day. The estimated mean monetary savings over a year were 190€ and 126€ per person, respectively (see Additional file 2).

#### One-way sensitivity analyses

**Fig. 2:**
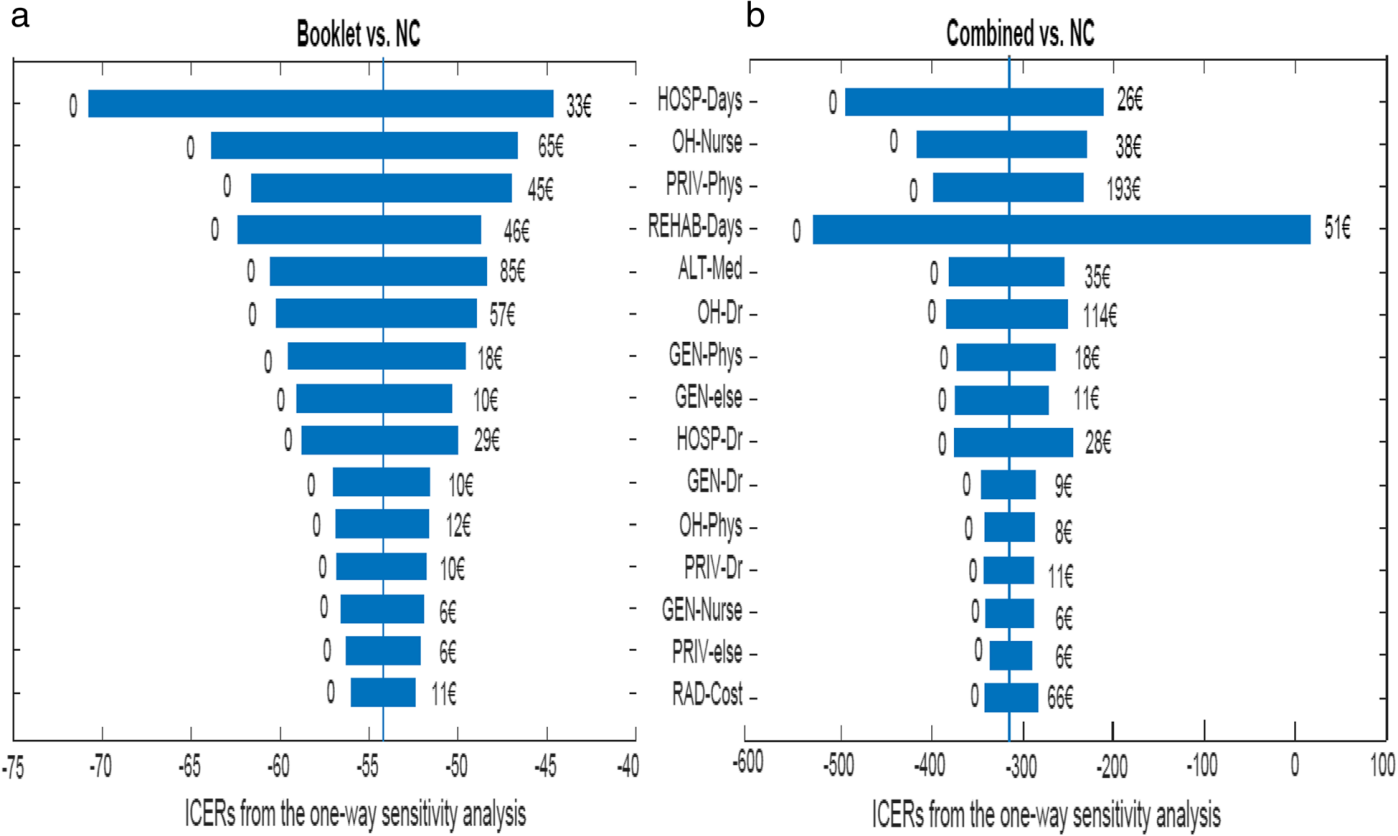
**a** and **b** One-way sensitivity analyses (tornado diagrams) for the comparisons of Booklet vs. NC (**a**) and Combined vs. NC (**b**). Horizontals bar indicate the change in every cost variable ranging from percentiles 5 to 95 %. The graph shows the influence of the change in one unit cost in the ICER (€ per sickness absence day), when other values remain at their base level. Around the ICER, bars may be asymmetric because of the Bayesian statistical procedure. In both graphs, variables were sorted according to their influence in the comparison of Booklet vs. NC (most influential variable on top). Variables OH-else, HOSP-Nurse, PRIV-Nurse, HOSP-Phys, HOSP-else and INTCOST have been deleted because of minimal effects in the ICER. The descriptions of all cost-items are shown in the Additional file 1 [Booklet, Back Book booklet group; Combined, Back Book with oral advice group; NC, natural course of LBP group]

Booklet vs. NC was not sensitive to any cost variable. The ICER varied from -71€ to -45€ per SA day avoided (Fig. 2a). In Combined vs. NC, however, the ICER varied from -530 to 15€ (Fig. 2b) meaning that the result was sensitive to a single expensive cost item (rehabilitation days).

#### Probabilistic sensitivity analysis

**Fig. 3:**
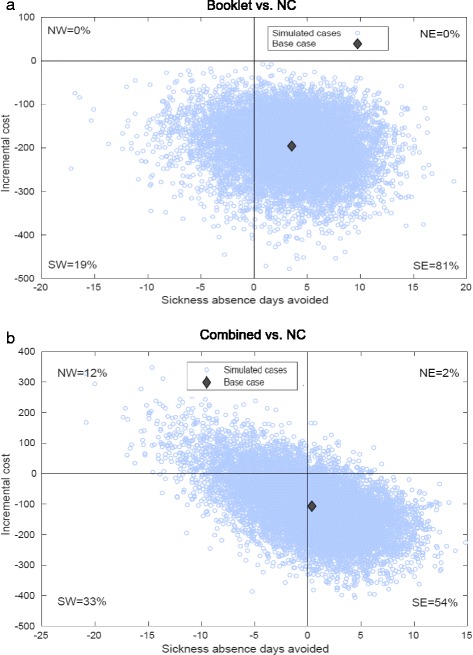
**a** and **b** Cost-effectiveness (CE) planes for the comparisons of Booklet (**a**) and Combined (**b**) vs. NC. Base case is indicated with a diamond and bootstrapped, simulated cases with small dots. Both graphs show the probability of the bootstrapped, simulated cases across the CE plane quadrants. In the north-eastern (NE) quadrant, intervention group is considered more costly and more effective than the control group and in the north-western (NW) quadrant, more costly and less effective, in the south-western (SW) less costly and less effective and in the south-eastern (SE) more effective and less costly, respectively. [See also Additional file 2]

For Booklet vs. NC, the mean incremental cost (with 95 % CI) was -196€ (-308 – -96), (negative figure indicates savings) and the mean incremental effect -3.5 (95 % CI -10 – 3.8), representing avoided work absence. According to the CE plane (Fig. 3a), the base case and also 81 % of the simulated cases were situated in the south-eastern (SE) quadrant, suggesting that the intervention was both cost saving and more effective (see Additional file 2). All bootstrapped, simulated cases were located below the horizontal line showing that the intervention clearly reduced HC costs.

According to CEAC, at any level of willingness to pay for a SA day avoided the probability of the Booklet intervention being acceptable is 81 % (for any positive cost of a SA day) (see Additional file 3).

For Combined vs. NC, the mean incremental cost (with 95 % CI) was -107€ (-258 – 61), (negative figure indicating savings), but the mean incremental effect was only marginal, -0.4 days (-7.5–7.8), representing avoided work absence. Although the base case was located in the SE quadrant suggesting more effectiveness and fewer costs, only about 54 % of the simulated, bootstrapped cases (Fig. 3b) fell in that quadrant (see also Additional file 2). However, about 87 % of the simulated cases lay in the two southern quadrants indicating reduced HC costs.

According to the CEAC, at any level of willingness to pay for a SA day avoided the probability of the Combined intervention being acceptable is between 62 % and 57 % (from zero cost to all costs above 200€) (see Additional file 3).

#### Sensitivity analysis using two data sets

The results and the conclusions drawn from the complete case analysis were largely comparable with the main analysis (see Additional file 2). Few differences were noticed, however. The Combined intervention was more cost-effective in the complete case analysis and the results were not that sensitive to one cost item as in the main analysis (data not shown).

### Sickness absence days

All cause SA days during two years were trend-wise lower in both intervention groups in comparison to NC (Table 2).

**Table 2:** Results of the study outcome variables

Outcomes/analysis	Combined		Booklet		NC		Total		Combined vs. NC			Booklet vs. NC		
	mean	SD	mean	SD	mean	SD	mean	SD	MD	95 % CI	p	MD	95 % CI	p
Main analysis^a^														
PHI	3.0	3.6	2.0	2.9	4.5	3.8	3.1	3.6	**-1.5**	**-2.8 – -0.3**	**0.01**	**-2.5**	**-3.8 – -1.3**	**0.00**
LBP	21	22	20	20	23	19	21	20	-2	-9 – 6	0.86	-3	-11 – 4	0.51
SA first year	12	22	12	18	16	30	13	24	-5	-13 – 4	0.39	-4	-13 – 4	0.56
SA second year	16	38	12	34	16	23	15	32	0	-12 – 11	1.00	-4	-15 – 8	0.74
SA total in two years	27	50	24	48	32	43	28	47	-5	-22 – 12	0.76	-8	-25 – 9	0.50
HC costs in 12 months, €	195	700	108	142	303	577	199	530	-108	-297 – 82	0.37	**-195**	**-382 – -7**	**0.04**
Complete case analysis^b^														
PHI	2.6	3.8	1.5	3.1	4.1	4.1	2.6	3.8	-1.4	-3.0 – 0.1	0.10	**-2.6**	**-4.2 – -0.9**	**0.00**
LBP	20	23	17	22	23	22	20	22	-2	-12 – 8	0.87	-5	-15 – 4	0.41
SA first year	13	25	12	19	13	20	13	22	0	-10 – 10	1.00	-2	-11 – 8	0.93
SA second year	16	42	13	39	17	23	15	37	-1	-17 – 15	0.99	-4	-20 – 12	0.84
SA two years	29	55	25	54	30	32	28	49	-1	-23 – 21	0.99	-5	-27 – 16	0.83
HC costs in 12 months, €	188	808	73	151	370	730	196	633	-182	-457 – 93	0.26	**-297**	**-572 – -22**	**0.03**

^a^Main analysis includes 264 participants in Combined (89), Booklet (92) and NC (83) groups

^b^Complete case analysis includes 185 participants in Combined (67), Booklet (67) and NC (51) groups

The main analysis includes multiply imputed data of 264 cases and complete case analysis includes the original, available data (*n* = 185). Table shows the means and standard deviations in all study groups and the group comparisons between intervention groups (Combined and Booklet) and control (*NC* natural course group). [Mean, standard deviation (SD), mean difference (MD), 95 % confidence interval (95 % CI), p-value of the group comparison, *PHI* Physical impairment by the Roland-Morris 18-item Disability Questionnaire (range 0–18), *LBP* low back pain in Visual Analogue Scale (VAS, range 0–100 mm), *SA* sickness absence (days), *HC* health care]

Figures are bolded when considered statistically significant

### Other health outcomes

According to the main analysis, the mean difference of PHI in Booklet vs. NC, two years from baseline, was -2.5 [95 % CI -3.8 – -1.3], and for Combined vs. NC -1.5 [95 % CI -2.8 – -0.3]. The LBP intensity tended to decrease in both arms, but the changes were not statistically significant (Table 2).

### Main findings

Booklet information was cost-effective in comparison to the natural course of LBP. In short, a delivery of simple written information reduced the direct health care costs and saved sickness absence days. Although the combination of booklet information and face-to-face advice also reduced the costs of health care (87 % probability), the effectiveness of the intervention on sickness absence was negligible. The cost-effectiveness analysis of the combined intervention was also sensitive to a single cost item. Physical impairment also decreased in both intervention groups over the two years.

### Strengths and weaknesses of the study

The main strengths of the study lie in the pragmatic OH setting, the inclusion of the natural course control group, and the long-term follow-up. Comprehensive and reliable sickness absence data are also an asset in this study. This quasi-experimental cost-effectiveness study was prepared alongside with our previous RCT [15]. Employees were invited to participate in the study on the basis of a health risk appraisal (response rate 71 %) that comprised items of LBP, sciatica and LBP history. All employees who were included in the study groups shared the same eligibility criteria and were primarily not sick-listed. Natural course of LBP (NC) group acted as the control for the interventions. NC was a random sample from the same cohort as the intervention groups and received no intervention. Although the study is based on a single industrial location, the study cohort aptly represents the general distribution of the Finnish workforce (age, gender, socio-economic class).

Generally, sickness absence serves as a measure of health in the working population when health is understood as a mixture of social, psychological and physiological functioning [31, 32]. The sickness absence data in this study were highly reliable also because the same data were used as a basis for the health insurance payments in the company.

The health care usage in our study may look very different from other health care systems. However, all public, private and occupational health care visits in our data may be considered as components of a uniform primary HC resource use. Considering the pragmatic study design, a real life OH organisation and comprehensive SA data, our results can be easily transferred to OH practice and to some extent to primary care, as well.

Some weaknesses were identified, though. The quasi-experimental study design may be considered as such, although we were able to include the NC group as a comparison, which – on the other hand – is a definite strength. The study groups were comparable as regards the basic characteristics (Table 1). However, the proportion of blue collar workers was slightly higher in the NC group and they assessed their work ability lower (borderline difference) than those in the intervention groups (the intervention goups were pooled for the comparison). However, since there were no differences in physical and mental workload or in several LB-specific variables (PHI, intensity of LBP, pain-related fear) prior to the study, pre-study sickness absence or LB history, NC is considered comparable with the intervention groups.

In contrast to a fairly good follow-up participation in the intervention groups (73–74 %), the response rate in the NC group was somewhat lower (61 %), which could potentially indicate selective participation and cause bias. All study participants were familiar with the study questionnaires, having already responded to the employee survey at the beginning of the study. NC group members were also able to respond to their questionnaires like their fellow participants. Hence, we do not recognise any systematic reason or occurrence that would explain the missed follow-up visits.

We have analysed the HC utilisation data from the time-period of 13–24 months in this study, because the NC group received only one follow-up questionnaire, scheduled at 24-months after the study started. The same questionnaire was used with all participants and it covered HC utilisation from the last 12 months. This may be considered as weakness, but we opted not to intervene in the NC group by any means during the follow-up of two years. We also thought that the HC utilisation recall period could not be longer than 12 months. We were aiming for secondary prevention of symptoms and were expecting long-term results, longer than just 12 months. Recall bias is considered similar in all groups, due to the uniform data collection.

Although study participants were advised to exclude their study visits from HC utilisation, in a few cases, some may have mistakenly included them. Because all study participants were working in the same company, the NC group may have been somewhat contaminated by the interventions. However, contamination or inclusion of study visits would more likely have diluted the differences between the intervention and control groups than increased them.

At first it seems surprising that the cost-effectiveness of combined patient information was weaker than the booklet information alone. Some characteristics about the verbal information might explain at least part of this controversy. According to Henrotin et al, patient information should be consistent even though it is delivered to patients in various methods [33] like verbal, written, video etc. Otherwise, distracted information may cause confusion among the patients and diminish the effect of the information. Verbal advice is very sensitive to inconsistency or disturbances per se. Physical and social environment of the patient and nurse, nurse-patient interaction, or intrapersonal characteristics can disturb the fragile connection between the patient and the health service provider. Other explanations can be that individuals in the Booklet group may have read the booklet more intensively than those in the Combined group and therefore, complied more closely with the content, or have used the booklet also later as a guideline.

### Methodological considerations

In our study, about 29 % of the study visits were missing after two years. Multiple imputation attenuated the cost-effectiveness results of the Booklet group and the results of the Combined group became less apparent (see Additional file 2). However, the main conclusions of the study remained the same, whether analysed with the imputed or the complete case data (Table 2). The follow-up visit activity was similar in both intervention groups.

CEA showed that results in the Combined group were sensitive to rehabilitation centre inpatient costs. Data show that the cost was due to a single inpatient episode of only one person. If this cost was neglected as an outlier, HC costs in the Combined would fall at around the same level as in the Booklet group. On the other hand, even though some high cost categories (rehabilitation centre days and hospital inpatient days) were neglected in the NC group, HC usage and costs would still remain high in the NC, because HC usage was higher in almost all HC categories in comparison with the intervention groups.

Because of the health care perspective in our study, we have omitted non-medical costs such as travel time, time expenses for the HC visits or out-of-pocket costs. As some previous studies have shown, the impact of these costs would have been minor, anyway.

Our cost data covers the whole study period of two years in the intervention groups. The data show that the total direct HC costs from the whole study period were about twice (data not shown) compared to the costs from the last 12-months’ time-sequence in the intervention groups. Therefore, we may estimate that the total 2-year costs would be two-fold in the NC group as well. Hence, the incremental cost were estimated as -392€ in the Booklet arm and -214€ in the Combined in two years, negative figure indicating savings.

### Comparison with previous studies

To the best of our knowledge, there are no parallel cost-effectiveness studies that would have randomly allocated mild LBP patients in the groups to whom they have provided information in the OH setting. Yet, information and advice have already shown to have positive effects on LBP specific outcomes or recovery, either alone [8, 10] or as an adjunct to other therapies [33–35] in various other settings.

Previous cost-effectiveness studies in primary care have focused on either acute sciatica [36] or chronic LBP [37, 38], i.e. more symptomatic patients [39], than in our study. In the majority of previous studies, patients have also been absent from work [40–42] at baseline. Participants have been recruited either from back clinics or other physician consultations, or invited to participate in the study on the basis of SA records. Primary care interventions for sub-acute or recurrent LBP have been cost-effective in many cases [43]. However, the usual care control group has not been consistent or properly defined in these studies. In addition, these interventions have generally been carried out by a physician or in collaboration with a physiotherapist and therefore, are not entirely comparable with our study.

In our previous RCT, we found no difference in the health-related outcomes between the two mild LBP patient information arms [15]. However, when compared with the natural course of LBP we found a consistent reduction in PHI and also HC costs in both patient information arms.

Some recent studies have shown that a LBP management strategy that is based on a patient-level risk-assessment (e.g. low, medium or high risk of LBP) in primary care is more efficient and cost-effective than a non-stratified approach [44–46]. Hill et al. [46] found that the interventions (patient information and physiotherapist consultations) were cost-effective for medium- and high-risk patients. The low-risk subgroup only received one patient information session (educational video and the Back Book). As a result, work loss decreased in the low risk intervention group in comparison to the control group (usual care). Although their recruitment strategy was different from ours, the main characteristics of the study participants in the low-risk group are comparable. Whitehurst et al. later analysed the results of the low-risk group [44] and found that the intervention was cost effective also in the low-risk subgroup.

### Clinical significance of the study

Due to our focus on mild LBP, PHI values were relatively low at baseline. Nevertheless, we discovered a small, yet significant, mean difference in the group comparisons against natural course of LBP. Although the effect sizes were modest in absolute figures, the proportional effects in both comparisons were 36–60 % of the corresponding baseline values. The results were also long-lasting [47]. Roland-Morris Disability Questionnaire is rather insensitive to change when symptom levels are low [48], suggesting that even minor differences may be of importance.

The study participants were recruited and the interventions were carried out in a pragmatic OH environment by an OH nurse. Our results indicate that the OH system can reconsider their professional roles and errands. In contrast to previous physician-led interventions, our nurse-guided patient information was cost-effective in mild LBP, and there was no need for the support of a physiotherapist or a physician. In the case of mild LBP, health examinations in the OH could be more targeted and perform a stratified, symptom-based, preventive approach. Our results indicate that a proactive, targeted LBP management with appropriate patient information leads to positive outcomes and reduced costs in the OH setting [49].

According to the health care usage (see Additional file 1), a vast majority of all primary care consultations (mainly by nurses and physicians) were performed at the OH service. Until present, OH service plays an important role in the primary health care of the Finnish working population. It also has resources and ability to bring secondary preventive actions into practice [50].

For the employees that reported mild LBP, simple patient information booklet was effective and cost-saving in comparison with the natural course of LBP. Further studies in this field should aim at larger patient samples, and introduce a genuine randomised design also concerning the control group.

## Conclusions

Low back specific patient information booklet was cost-effective in comparison with the natural course of LBP over two years. Booklet intervention with additional face-to-face information resulted in lower health care costs, but the effectiveness in sickness absence was minimal. Physical impairment decreased in both intervention arms. Simple booklet information is beneficial for employees who report mild LBP in the OH setting, and also saves costs of the health care system.

### Study ethics

The South Karelian Central Hospital Research Ethics Board approved the study on 13.09.2001. All participants received written information regarding the study, in accordance with the Declaration of Helsinki. Only the participants who gave their signed informed consent were included in the study. The documents above are stored with the other study material.

## Additional files

Additional file 1: Health care (HC) resource usage and direct HC costs in all study groups (with the number of participants indicated) during the last 12 months of the two-year follow-up according to available data (data not imputed). Number of units per group, unit cost, total cost per group and mean cost (per participant) with standard deviation (SD). Subtotals show units and costs in some basic HC categories. Zero unit/cost lines have been deleted. [HC, health care; OH, Occupational health; Booklet, Back Book group; Combined, Back Book and advice group; NC, Natural course group; PT, physiotherapist; OPR, other professional]. (DOCX 209 kb) Additional file 2: The results of two cost-effectiveness analyses (CEA), based on the multiply imputed data (main analysis) and the complete case analysis (original data). Table also shows the mean monetary savings per person in the group comparisons of the intervention groups (Combined and Booklet) against the control (NC = natural course of LBP group) as well as the distribution of bootstrapped, simulated cases across the CE plane qvadrants (in percentages). [mean; 95%CI = 95 % confidence interval; ICER = incremental cost-effectiveness ratio; incremental costs and effects]. (DOCX 202 kb) Additional file 3: Cost-effectiveness acceptability curves (CEAC) of the comparisons Booklet vs. NC (dotted line) and Combined vs. NC (solid line). CEAC line indicates the probability that the intervention is cost-effective in comparison to NC (natural course of mild LBP) when the maximum acceptable cost per SA day is 200€ [Booklet, Back Book booklet group; Combined, Back Book with oral advice group; CEA, cost-effectiveness analysis]. (DOCX 49 kb)

## Abbreviations

Booklet: booklet alone patient information arm
CE: cost-effectiveness
CEA: cost-effectiveness analysis
CEAC: cost-effectiveness acceptability curve
Combined: booklet and verbal advice patient information arm
HC: health care
IC: incremental cost
ICER: inceremental cost-effectiveness ratio
IE: incremental effect
LB: low back
LBP: low back pain
NC: natural course control group
PHI: physical impairment
SA: sickness absence days
SD: standard deviation
