# Supplementary material for: Cost-effectiveness of providing patients with information on managing mild low-back symptoms in an occupational health setting
Source: BMC Public Health. 2016 Apr 12;16:316. doi: 10.1186/s12889-016-2974-4 (PMC4828818; doi:10.1186/s12889-016-2974-4)
Supplement: Additional file 1: — Health care (HC) resource usage and direct HC costs in all study groups (with the number of participants indicated) during the last 12 months of the two-year follow-up according to available data (data not imputed). Number of units per group, unit cost, total cost per group and mean cost (per participant) with standard deviation (SD). Subtotals show units and costs in some basic HC categories. Zero unit/cost lines have been deleted. [HC, health care; OH, Occupational health; Booklet, Back Book group; Combined, Back Book and advice group; NC, Natural course group; PT, physiotherapist; OPR, other professional]. (DOCX 209 kb) [file 12889_2016_2974_MOESM1_ESM.docx]

Additional file 1. Health care (HC) resource usage and direct HC costs in all study groups (with the number of participants indicated) during the last 12 months of the two-year follow-up according to available data (data not imputed). Number of units per group, unit cost, total cost per group and mean cost (per participant) with standard deviation (SD). Subtotals show units and costs in some basic HC categories. Zero unit/cost lines have been deleted. [HC, health care; OH, Occupational health; Booklet, Back Book group; Combined, Back Book and advice group; NC, Natural course group; PT, physiotherapist; OPR, other professional].
