# Supplementary material for: Cost-effectiveness of providing patients with information on managing mild low-back symptoms in an occupational health setting
Source: BMC Public Health. 2016 Apr 12;16:316. doi: 10.1186/s12889-016-2974-4 (PMC4828818; doi:10.1186/s12889-016-2974-4)
Supplement: Additional file 2: — The results of two cost-effectiveness analyses (CEA), based on the multiply imputed data (main analysis) and the complete case analysis (original data). Table also shows the mean monetary savings per person in the group comparisons of the intervention groups (Combined and Booklet) against the control (NC = natural course of LBP group) as well as the distribution of bootstrapped, simulated cases across the CE plane qvadrants (in percentages). [mean; 95%CI = 95 % confidence interval; ICER = incremental cost-effectiveness ratio; incremental costs and effects]. (DOCX 202 kb) [file 12889_2016_2974_MOESM2_ESM.docx]

Additional file 2. The results of two cost-effectiveness analyses (CEA), based on the multiply imputed data (main analysis) and the complete case analysis (original data).

Table also shows the mean monetary savings per person in the group comparisons of the intervention groups (Combined and Booklet) against the control (NC=natural course of LBP group) as well as the distribution of bootstrapped, simulated cases across the CE plane qvadrants (in percentages). [mean; 95%CI=95 % confidence interval; ICER=incremental cost-effectiveness ratio; incremental costs and effects]
