# Supplementary material for: Cost-effectiveness of providing patients with information on managing mild low-back symptoms in an occupational health setting
Source: BMC Public Health. 2016 Apr 12;16:316. doi: 10.1186/s12889-016-2974-4 (PMC4828818; doi:10.1186/s12889-016-2974-4)

Additional file 3. Cost-effectiveness acceptability curves (CEAC) of the comparisons Booklet vs. NC (dotted line) and Combined vs. NC (solid line). CEAC line indicates the probability that the intervention is cost-effective in comparison to NC (natural course of mild LBP) when the maximum acceptable cost per SA day is 200€ [Booklet, Back Book booklet group; Combined, Back Book with oral advice group; CEA, cost-effectiveness analysis].


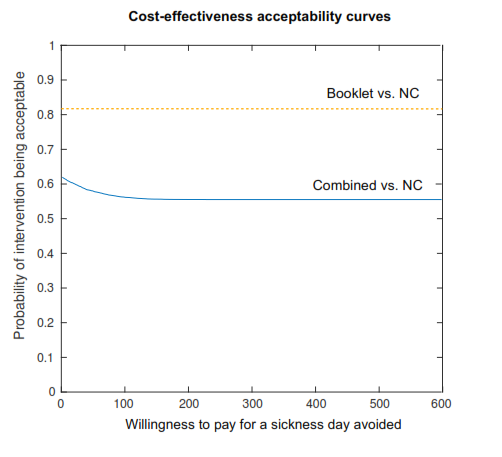

Supplement: Additional file 3: — Cost-effectiveness acceptability curves (CEAC) of the comparisons Booklet vs. NC (dotted line) and Combined vs. NC (solid line). CEAC line indicates the probability that the intervention is cost-effective in comparison to NC (natural course of mild LBP) when the maximum acceptable cost per SA day is 200€ [Booklet, Back Book booklet group; Combined, Back Book with oral advice group; CEA, cost-effectiveness analysis]. (DOCX 49 kb) [file 12889_2016_2974_MOESM3_ESM.docx]
